# Parametric and cadaveric models of lumbar flexion instability and flexion restricting dynamic stabilization system

**DOI:** 10.1007/s00586-013-2934-y

**Published:** 2013-08-17

**Authors:** Louis C. Fielding, Todd F. Alamin, Leonard I. Voronov, Gerard Carandang, Robert M. Havey, Avinash G. Patwardhan

**Affiliations:** 1 Simpirica Spine, Inc., San Carlos, CA USA; 2 Department of Orthopaedic Surgery, Stanford University School of Medicine, San Carlos, CA USA; 3 Musculoskeletal Biomechanics Research Laboratory, Loyola University Chicago and Edward Hines Jr. VA Hospital, Fifth Avenue & Roosevelt Road, Hines, IL 60141 USA

**Keywords:** Biomechanics, Dynamic stabilization, Flexion, Instability, Parametric model

## Abstract

**Purpose:**

Development of a dynamic stabilization system often involves costly and time-consuming design iterations, testing and computational modeling. The aims of this study were (1) develop a simple parametric model of lumbar flexion instability and use this model to identify the appropriate stiffness of a flexion restricting stabilization system (FRSS), and (2) in a cadaveric experiment, validate the predictive value of the parametric model.

**Methods:**

Literature was surveyed for typical parameters of intact and destabilized spines: stiffness in the high flexibility zone (HFZ) and high stiffness zone, and size of the HFZ. These values were used to construct a bilinear parametric model of flexion kinematics of intact and destabilized lumbar spines. FRSS implantation was modeled by iteratively superimposing constant flexion stiffnesses onto the parametric model. Five cadaveric lumbar spines were tested intact; after L4–L5 destabilization (nucleotomy, midline decompression); and after FRSS implantation. Specimens were loaded in flexion/extension (8 Nm/6 Nm) with 400 N follower load to characterize kinematics for comparison with the parametric model.

**Results:**

To accomplish the goal of reducing ROM to intact levels and increasing stiffness to approximately 50 % greater than intact levels, flexion stiffness contributed by the FRSS was determined to be 0.5 Nm/deg using the parametric model. In biomechanical testing, the FRSS restored ROM of the destabilized segment from 146 ± 13 to 105 ± 21 % of intact, and stiffness in the HFZ from 41 ± 7 to 135 ± 38 % of intact.

**Conclusions:**

Testing demonstrated excellent predictive value of the parametric model, and that the FRSS attained the desired biomechanical performance developed with the model. A simple parametric model may allow efficient optimization of kinematic design parameters.

## Introduction

Flexion is the most significant motion of the lumbar spine: it involves the greatest range of motion (ROM) [1, 2] and is the most exercised during activities of daily living [3, 4]. As such, lumbar instability in flexion is of clinical significance. Instability in flexion is associated with degenerative pathology such as early degenerative disc disease (DDD) [5–7] and degenerative spondylolisthesis (DS) [8, 9] as well as decompression surgery due to resection of posterior structures [10, 11]. Instability in flexion may be exhibited at any level of the lumbar spine; however, instability at the L4–L5 level is most prevalent [12–14].

Flexion instability may be defined as a symptomatic increase in the flexion ROM, as well as a symptomatic decrease in stiffness within the high flexibility zone (HFZ)—the range in which large motions are effected with minimal effort [15, 16], and in which most activities of daily living occur [4, 15]. The symptoms associated with flexion instability may either be pain or a recurrence of neurocompressive symptoms. Flexion is also known to be coupled to segmental translation [17–19] and therefore instability in flexion may be coupled with translational instability. This may be of particular interest in patients with DS.

A flexion-restricting stabilization system (FRSS) has been proposed to address this specific biomechanical pathology. Defining biomechanical parameters of a dynamic stabilization device often requires iterative prototyping, testing, and computational modeling—costly, time-consuming and resource-intensive approaches. To facilitate iteration and development of the biomechanical requirements of the FRSS, a simple parametric model was developed to predict segmental kinematics, as a function of the inherent segmental biomechanical properties and the mechanical properties of the FRSS.

The purpose of this experiment was twofold: (1) utilize the parametric model to identify the appropriate segmental flexion-bending stiffness to be provided by the FRSS; and (2) in a cadaveric experiment, validate the effectiveness of the parametric model to predict the biomechanical effect of simulated degenerative and iatrogenic injury of the type resulting in flexion instability, and the effect of implantation of the FRSS on the destabilized spine.

## Materials and methods

### Parametric model

**Fig. 1:**
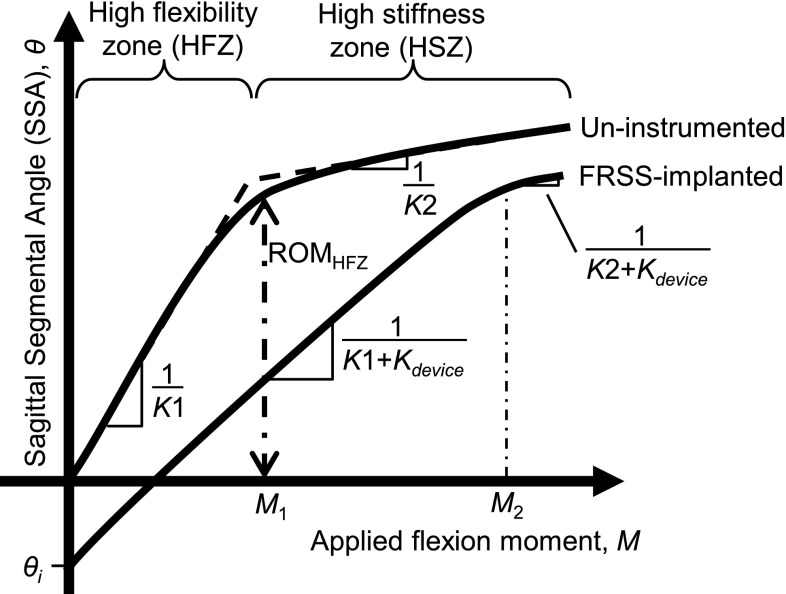
Characteristic bi-linear flexion loading behavior of spinal motion segments

The spine is known to exhibit substantially bi-linear mechanical behavior (Fig. 1) [20]. The HFZ, characterized by low flexion stiffness, permits functional motion and activity without requiring excessive muscular effort. Outside of the HFZ, segmental stiffness dramatically increases in the high stiffness zone (HSZ). Degenerative pathology or surgical intervention may result in laxity, i.e. decreased stiffness within the HFZ and increased HFZ ROM, in addition to increased total flexion ROM [11, 21].

**Fig. 2:**
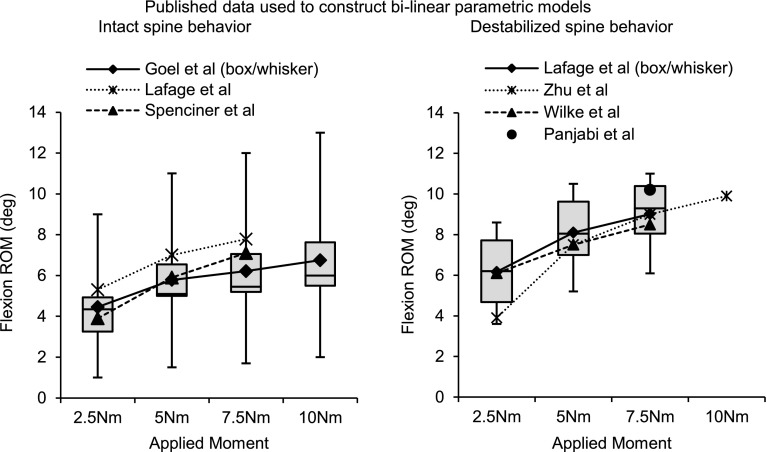
Published intact and destabilized spine behavior used to construct parametric model

To determine the appropriate flexion bending stiffness to be provided by the FRSS, published biomechanical literature was surveyed and data abstracted to identify typical values for the flexion stiffness within the HFZ and HSZ (K1 and K2) as well as the extent of the HFZ (ROM_HFZ_), for both intact and destabilized cadaveric specimens (Table 1; Fig. 2) [10, 11, 17, 20, 22, 23]. These typical values were then used to construct a bilinear lumped-parameters model of L4–L5 flexion-bending characteristics, which can be plotted as segmental sagittal angle (SSA) versus applied moment. The bilinear model may be summarized as

$${\text{for}}\,0 < M \le M_{1} :\theta = \frac{M}{{K_{1} }}$$

$${\text{and}}\,{\text{for}}\,M > M1:\theta = {\text{ROM}}_{\text{HFZ}} + \frac{{M - M_{1} }}{{K_{2} }},$$

where

$$M_{1} = K_{1} \cdot {\text{ROM}}_{\text{HFZ}} ,$$

**Fig. 3:**
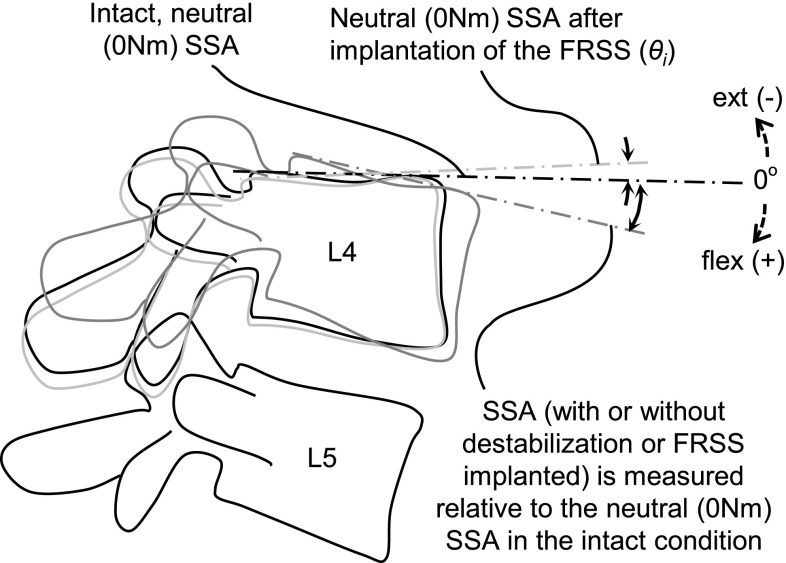
Segmental sagittal angle (SSA) is an absolute measurement of the sagittal angulation of L4 with respect to L5, and is measured relative to the neutral (0 Nm), intact condition

*M* is the applied flexion moment (Nm), *θ* is the SSA relative to the intact, neutral (0 Nm) condition (Fig. 3), and *M*_1_ is the applied flexion moment to achieve the ROM_HFZ_.

**Table 1:** Typical flexion bending stiffness abstracted from previously reported literature [10, 11, 17, 20, 22, 23]

	K_1_ (Nm/deg)	K_2_ (Nm/deg)	ROM_HFZ_
Intact	0.6	2.5	6°
Destabilized	0.4	2.3	8°

**Table 2:** L4–L5 segmental rotation results (mean ± SD) and statistical comparisons

	1. Intact	*p* ^1^ 1 vs. 2	2. Destabilized	*p* ^1^ 2 vs. 3	3. Destab + FRSS	*p* ^1^ 3 vs. 1	*p* ANOVA
Total flexion–extension ROM	10.7° ± 1.5°	<0.01	15.5° ± 1.4°	<0.01	11.2° ± 2.2°	0.32	<0.01
Maximum SSA (8 Nm flexion)	7.5° ± 1.1°	<0.01	12.6° ± 1.8°	<0.01	6.9° ± 2.0°	0.27	<0.01
Lateral bending ROM	9.2° ± 2.3°	<0.01	12.1° ± 3.2°	0.13	11.3° ± 3.5°	0.08	<0.01
Axial rotation ROM	3.0° ± 1.2°	<0.01	4.9° ± 1.5°	0.01	3.7° ± 1.8°	0.28	<0.01

^1^Post hoc 1-tailed *t* test with Bonferroni’s correction for multiple comparisons

To model the application of the FRSS, constant flexion stiffness was superimposed on the bilinear approximation, as well as an offset due to the initial implantation tension of the FRSS. Thus, with the addition of the FRSS the bilinear model is expressed as

$${\text{for}}\,0 < M \le M_{1} :\theta = \theta_{i} + \frac{M}{{K_{1} + K_{\text{device}} }}$$

$${\text{and}}\,{\text{for}}\,M > M_{2} :\theta = {\text{ROM}}_{\text{HFZ}} + \frac{{M - M_{2} }}{{K_{2} + K_{\text{device}} }}$$

where

$$M_{2} = (K_{1} + K_{\text{device}} )({\text{ROM}}_{\text{HFZ}} - \theta_{i} ),$$

*K*_device_ is the flexion-bending stiffness provided by the device (Nm/deg), *θ*_*i*_ is the initial change in SSA (in degrees) after implantation of the device, relative to the intact neutral condition, and *M*_2_ is the flexion moment for the implanted segment to achieve the ROM_HFZ_. For both the uninstrumented and FRSS-implanted spine, ROM_HFZ_ was considered to remain constant as this property would depend on segmental tissue strains. Thus, the effort required to achieve ROM_HFZ_ for the implanted spine (*M*_2_) increases with the added flexion stiffness provided by the FRSS.

### Biomechanical validation testing

Biomechanical flexibility testing was performed on cadaveric specimens to validate the predictive value of the model, and that the FRSS attained the desired performance predicted by the model.

#### Specimens

Five (5) fresh-frozen human lumbar spines (L1–S1; age range 27–64 years) were tested. Specimens had no previous spinal surgery and no radiographic evidence of significant pathology. After thawing, specimens were cleared of extraneous soft tissue (leaving the discs, facet joints, and ligaments intact) and the L1 vertebra and sacrum were anchored in cups using PMMA and screws.

#### Mechanical flexibility testing

**Fig. 4:**
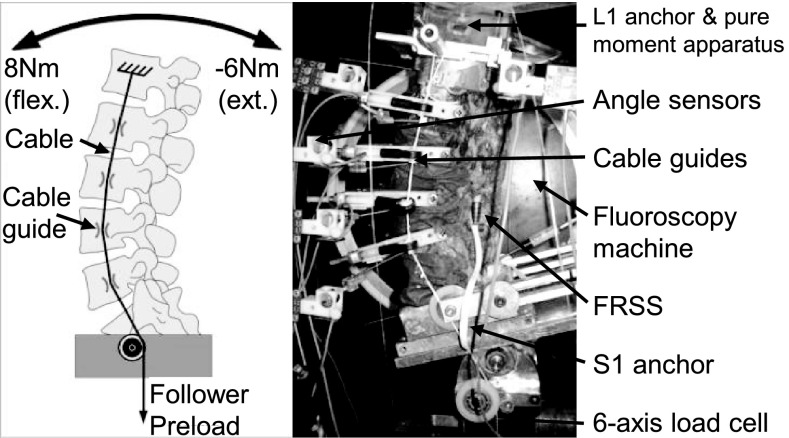
Schematic and photograph of test setup with follower load

All tests were performed at room temperature. Care was taken to prevent dehydration of the tissue by wrapping the specimens in saline-soaked gauze. The follower load technique was used to apply a compressive preload to the lumbar spine during the ROM experiments in flexion and extension and has been previously described [20, 22] (Fig. 4).

The load–displacement behavior was quantified for the ROM from extension (−6 Nm applied moment) to flexion (8 Nm), while under a 400 N compressive follower preload. The 400 N preload was selected as representative of trunk weight and muscle activation forces [24]. In addition to flexion–extension, ROM was also measured for lateral bending (±6 Nm) and axial rotation (±5 Nm) in pure moment loading (no follower preload) for characterization purposes. These applied moment values were chosen to test the specimen to comparable maximum in vivo loads without damaging anatomic structures [25]. The load–displacement data were collected repeatedly until two reproducible load–displacement loops were obtained. This generally required a maximum of three loading cycles.

The motion of the L1, L2, L3, L4, and L5 vertebrae relative to the sacrum were measured using an optoelectronic motion measurement system (Model 3020, Optotrak^®^, Northern Digital, Waterloo, ON, Canada). In addition, bi-axial angle sensors (Model 902-45, Applied Geomechanics, Santa Cruz, CA, USA) were mounted on each vertebra to allow real-time feedback for the optimization of the preload path.

During flexion and extension testing, lateral fluoroscopic images were captured in the extension (−6 Nm), neutral (0 Nm) and flexion (8 Nm) loading conditions using a digital video fluoroscopy machine (OEC 9800 Plus, GE OEC Medical Systems, Inc., Salt Lake City, UT, USA). A six-component load cell (Model MC3A-6-250, AMTI Multi-component transducers, AMTI Inc., Newton, MA, USA) placed under the specimen measured the applied compressive preload and moments.

#### Destabilization

**Fig. 5:**
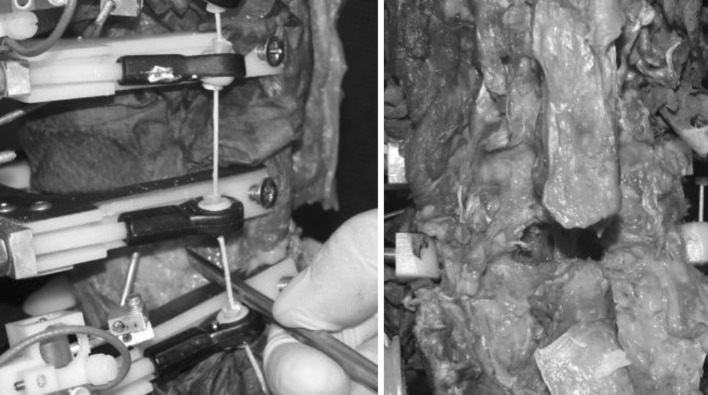
Destabilization: denucleation (*left*) and midline decompression (*right*)

Following testing in the intact condition, all spines were surgically destabilized at L4–L5 with a midline decompression involving resection of the interspinous/supraspinous ligament complex, a portion of the laminae and spinous processes of L4 and L5, and bilateral partial medial facetectomies, as well as total denucleation through a posterolateral incision in the annulus. The destabilizations used in this study simulated both degenerative and surgically induced instabilities, and are consistent with destabilization models used in previously published biomechanical research [10, 11, 22, 23, 26]. The midline decompression performed in this experiment was typical of a standard lumbar decompression, and the denucleation was intended to simulate the effect of nuclear dehydration associated with disc degeneration (Fig. 5). The index level for destabilization and device implantation was L4–L5 for all specimens. L4–L5 was selected as it is the most prevalent level for DDD and DS [8, 12–14].

#### FRSS implantation

**Fig. 6:**
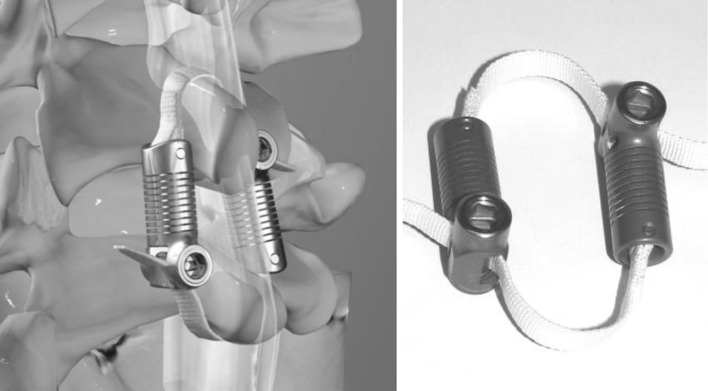
Rendering and photograph of the FRSS

The FRSS (LimiFlex™ Spinal Stabilization System, Simpirica Spine, San Carlos, CA, USA) comprises a pair of dynamic titanium rods secured to ultra-high molecular weight polyethylene (UHMWPE) straps with a roller-screw strap locking mechanism (Fig. 6). The straps loop around the cranial and caudal spinous processes of the treated segment to restrict segmental flexion. The device is tensioned and secured using instruments that allow for consistent tensioning, applying a nominal preload that induces slightly increased lordosis of the treated segment.

#### Data acquisition and analysis

 Segmental motion versus applied load data were acquired from the data acquisition systems of the test setup as described above. These data were normalized by the radiographically measured change in sagittal alignment to consistently obtain absolute sagittal segmental angle (SSA) data, relative to intact, across tested conditions. The SSA is an absolute measurement, and refers to the sagittal angulation of L4 with respect to L5 (Fig. 3). For consistency across tests and to track the postural effect of destabilization and FRSS implantation, SSA is measured relative to the neutral (0 Nm) condition under 400 N preload in the intact condition. Flexion stiffness within the HFZ and HSZ was calculated by linear regression using Microsoft Excel 2003 (Microsoft Corp., Remond, WA, USA). The size of the HFZ was taken from the knee portion of the loading curve nearest the intersection of the HFZ and HSZ regression lines (Fig. 1).

The lateral radiographs were used to measure absolute disc angle at neutral, which was used to track SSA across tests, as described above. The radiographs were further analyzed to measure segmental sagittal translation of the posterior aspect of the L4 inferior endplate with respect to the L5 superior endplate as described by White and Panjabi (Fig. 5–61, p. 354) [1]. Radiographic measurements of elongation of the dynamic titanium rods were used to estimate device loads based on the stiffness of the dynamic rods. Radiographic analysis was performed by a radiographic core laboratory using the QMA™ process (Medical Metrics, Houston, TX, USA). Data were tested for normality using the Kolmogorov–Smirnov test. Statistical comparison of the intact, destabilized and implanted conditions was performed using analysis of variance (ANOVA). Post-hoc tests for individual comparisons between groups were performed using 1-tailed *t* tests with Bonferroni’s corrections for multiple comparisons. A significance level of *p* ≤ 0.05 was used.

## Results

### Parametric kinematic model of spinal segment and FRSS

**Fig. 7:**
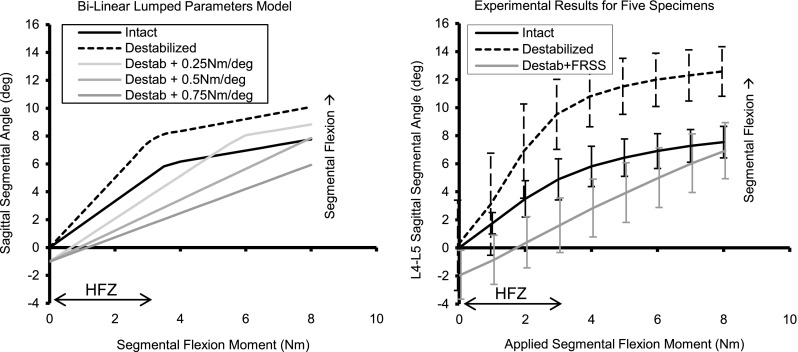
*Left* parametric model of intact and destabilized spines, and superposition of three stiffnesses for the FRSS. *Right* experimental results for five specimens

Different flexion-bending stiffnesses *K*_device_ proposed to be provided by the FRSS (e.g., 0.25, 0.5 and 0.75 Nm/deg) were iteratively superimposed on to the bilinear parametric model of the destabilized spines and compared to the intact model (Fig. 7). The surgical technique was developed to implant the FRSS with a consistent nominal pre-tension based on the device stiffness, moment arm, and HFZ stiffness of the destabilized segment. For implantation consistency the pre-tension was selected to apply a 0.5–1.0 Nm extension moment and bias the segment toward lordosis; thus, a −1° offset *θ*_*i*_ (toward extension) was included in the superposition model. The appropriate incremental segmental flexion stiffness to be provided by the FRSS was determined to be 0.5 Nm/deg, to reduce flexion ROM to intact levels, and increase stiffness to greater than intact (Fig. 7), such that the injured segment would have physiologic mobility yet not preferentially flex during normal activities. When solved for the tensile load borne by the device, the parametric model predicted the FRSS would experience a 75 N tensile load with the maximum 8 Nm flexion bending moment applied to a destabilized spine.

### Biomechanical validation testing

#### Range of motion and SSA

**Fig. 8:**
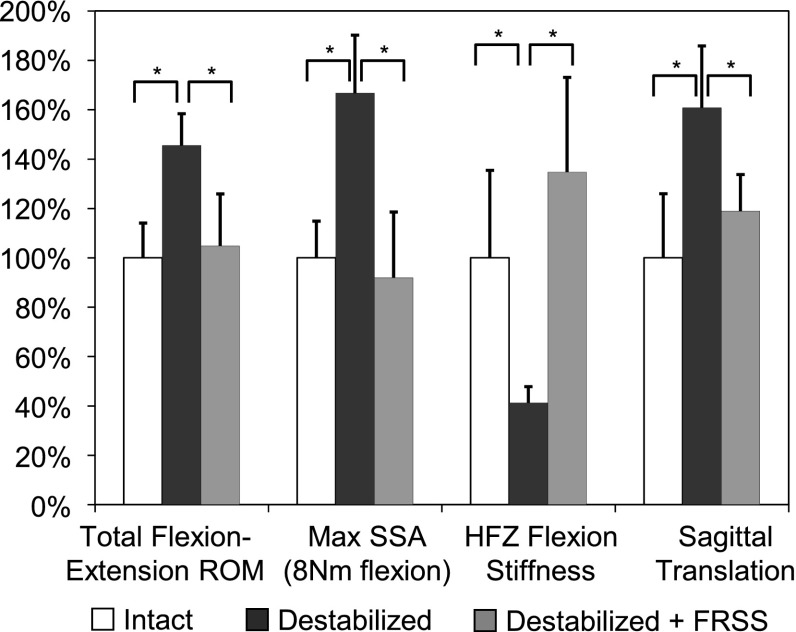
Relative effect of destabilization and FRSS implantation on sagittal motion parameters (*asterisks* denotes statistically significant difference)

The results and significance levels for segmental ROM and SSA are summarized in Table 2. Destabilization increased total flexion–extension ROM to 146 ± 13 % (mean ± SD) relative to the intact condition and increased the maximum L4–L5 SSA (corresponding to 8 Nm) to 167 ± 24 % of intact. Implantation of the FRSS at the destabilized segment reduced total flexion–extension ROM to 105 ± 21 % of intact and reduced the maximum SSA to 92 ± 27 % of intact. The instrumented values for total ROM and maximum SSA of the destabilized segments with FRSS implanted were not significantly different from the intact condition. Destabilization increased ROM in lateral bending to 132 ± 35 % and axial rotation to 164 ± 51 % of the intact values. FRSS implantation reduced lateral bending and axial rotation ROM to 123 ± 38 and 125 ± 62 % of the intact values, respectively (Figs. 7, 8).

#### HFZ flexion stiffness, HFZ ROM, segmental translation and FRSS load

**Fig. 9:**
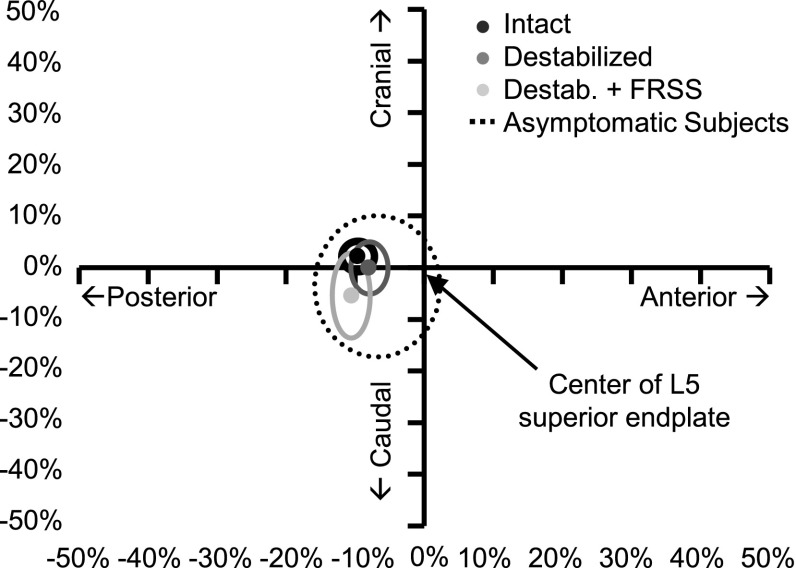
COR location with respect to the center of L5 superior endplate (mean and standard deviation shown; normalized to L5 superior endplate length); 95 % confidence interval for 75 asymptomatic subjects is shown for Ref. [19]

Quality of motion results for HFZ stiffness and ROM; segmental translation; and loading experienced by the FRSS are summarized in Table 3. Destabilization decreased HFZ flexion stiffness to 41 ± 7 % of intact; increased HFZ ROM to 177 ± 27 %; and increased segmental translation to 161 ± 25 % of the intact condition. Implantation of the FRSS at the destabilized level increased HFZ flexion stiffness to 135 ± 38 % of intact; reduced HFZ ROM to 113 ± 17 %; and reduced segmental translation to 119 ± 15 % of intact (Fig. 8). Flexion stiffness, HFZ ROM and segmental translation in the final implanted condition were not significantly different from intact. Destabilized specimens implanted with the FRSS displayed nearly linear load/displacement behavior without noticeable laxity, making identification of a knee to determine HFZ ROM difficult. Center of rotation (COR) moved anteriorly with destabilization and back posteriorly with FRSS implantation. There was a trend toward the COR moving caudally with both destabilization and FRSS implantation, however, for all conditions tested, COR was within the range reported for asymptomatic subjects [19], remaining just posterior to the center of the L5 superior endplate (Fig. 9). Based on the radiographic measurements of elongation of the dynamic titanium rods, the peak tensile loads experienced by the FRSS were found to be 73.6 ± 13.3 N (range 50.2–82.0 N).

**Table 3:** HFZ stiffness, ROM, translation and COR results (mean ± SD) and comparisons

	1. Intact	*p* ^1^ 1 vs. 2	2. Destabilized	*p* ^1^ 2 vs. 3	3. Destab + FRSS	*p* ^1^ 3 vs. 1	*p* ANOVA
HFZ stiffness (Nm/deg)	0.61 ± 0.22	0.02	0.25 ± 0.04	0.01	0.82 ± 0.23	0.32	<0.01
HFZ ROM	5.2° ± 0.7°	0.01	9.2° ± 1.4°	0.03	5.9° ± 0.9°	0.59	<0.01
Translation (mm)	1.5 ± 0.4	0.01	2.4 ± 0.4	0.01	1.8 ± 0.2	0.28	<0.01
COR, A/P (mm)^2^	−3.0 ± 0.6	0.04	−2.4 ± 0.8	0.06	−3.3 ± 0.7	0.79	0.05
COR, axial (mm)^3^	0.7 ± 0.9	0.47	0.0 ± 1.6	0.07	−1.7 ± 2.4	0.09	0.03

^1^Post hoc 1-tailed *t* test with Bonferroni’s correction for multiple comparisons

^2^Anterior–posterior distance from center of L5 endplate; anterior >0

^3^Axial distance from L5 endplate; cranial >0

## Discussion

In this experiment, we identified the appropriate flexion bending stiffness to be provided by the FRSS. Parametric modeling of lumbar spinal segments based on published biomechanical research allowed efficient iteration of design parameters prior to more resource-intensive modeling and prototyping. The parametric model identified 0.5 Nm/deg as the increase in segmental bending stiffness required to attain the biomechanical design objectives of the FRSS: restore the ROM of a destabilized segment to intact levels and increase the HFZ bending stiffness to more than intact. The clinical intent is that this behavior would permit a physiologic functional ROM, while stiffening the affected segment such that it would not preferentially flex relative to adjacent levels during moderate activities.

The FRSS was then designed according to these parameters. The tensile stiffness of the device was chosen to provide an incremental segmental bending stiffness of approximately 0.5 Nm/deg when secured to the spinous processes. Spinous process tension band fixation was chosen to avoid the invasiveness of pedicle screw fixation, while still being compatible with standard midline decompression techniques. In addition, spinous process fixation provides a longer lever arm relative to the COR in the sagittal plane as compared to transpedicular instrumentation, resulting in lower device forces as predicted by the parametric model and validated in the biomechanical testing.

Biomechanical validation testing demonstrated the kinematic behavior predicted by the parametric model. The biomechanical parameters for intact specimens tested here were very consistent with the typical values for intact specimens seen in the published studies that were used to construct the parametric model and design the FRSS. The destabilized specimens in this experiment displayed lower flexion bending stiffness and greater ROM than the typical published values used in the destabilized model. This may be due to the extent of the destabilization, which included complete denucleation and an extensive midline decompression. This may also be an artifact of specimen variability as well as heterogeneous loading conditions of the published reference data (Fig. 2). However, the qualitative effect of the destabilization was very consistent with that predicted by the model: increased HFZ and total flexion ROM and decreased HFZ flexion stiffness.

The FRSS achieved the desired design objectives of reducing ROM of a destabilized segment to intact levels. The parametric model used to design the FRSS identified 0.5 Nm/deg as the target segmental bending stiffness to be provided by the device, and the experimental results demonstrated that the FRSS increased bending stiffness of the destabilized segments by an average of 0.57 Nm/deg. Qualitatively, the FRSS linearized the kinematic profile of the lax destabilized segment, consistent with the model. The 73.6 N average peak tensile load (82 N max) experienced by the implant was consistent with the 75 N predicted by the parametric model. The relatively low loads experienced by the FRSS (and thus exerted on the anatomy) are a result of both the compliance of the FRSS and the lever arm afforded by the spinous processes.

Sagittal plane instability in flexion–extension has been associated with disc degeneration [5–7], degenerative spondylolisthesis [8, 9], and decompression surgery [10, 11, 22, 23]. Thus, flexion instability may result from either degenerative pathology or surgical intervention. Some studies have found that flexion may transmit loads to the intervertebral disc that may further exacerbate degeneration [27, 28]; in this case, flexion and biomechanical instability may constitute a positive feedback loop.

The FRSS was developed specifically to stabilize in flexion. While instability in other planes may be present, flexion involves the greatest ROM [1, 2] and is the most exercised during activities of daily living [3, 4]. The clinical hypothesis is that providing sagittal postural stabilization may provide durable clinical benefits. The fixation required to stabilize in a single plane may also avoid compound loading on the implant construct and fixation points. The reduction in axial rotation and trend toward small reduction in lateral bending ROM provided by the FRSS are likely due to increased facet engagement.

Previous studies have shown a coupled relationship between flexion/extension and intersegmental sagittal translation [17–19]. This experiment demonstrated that this coupled relationship is maintained through sequential destabilization and restabilization with the FRSS, such that restricting flexion ROM of a destabilized segment resulted in a concurrent, proportional reduction in segmental translation. The most significant implication of this finding is that translational instability such as that seen in degenerative spondylolisthesis may be addressed through restricting flexion.

For all tested conditions, the COR remained within the range reported for asymptomatic subjects [19]. This is consistent with the finding that the coupled relationship between flexion and translation was maintained through test conditions, as a large change in COR would affect this relationship. The small trend toward a caudal shift in COR may be due to lost disc height from the denucleation component of the destabilization.

Development of appropriate parameters and specifications for dynamic stabilization implants often involves time-consuming and costly iterations. Previous reports have described using finite element analyses (FEA) or iterative prototyping and testing to identify optimum implant properties [29]. The simple parametric model presented here represents an effective and efficient method to optimize kinematic parameters of a dynamic stabilization device. FEA and in vitro testing still retain critical roles in implant development. In vitro testing is an essential component of design validation. FEA techniques may allow analysis of properties that are difficult or unreliable to measure. Utilization of a simple, efficient parametric model allows rapid design optimization such that resources for intensive modeling or testing may be applied in a focused manner to an optimized implant or basic research.

Limitations of this study are that it is specific to static flexion load-deformation behavior. The model was developed for simulation of a uni-axial device that elastically constrains flexion. Therefore, commonly reported parameters such as the Neutral Zone [30] that relate to segmental hysteresis and viscoelastic properties are not addressed in this model. Because the FRSS is designed to specifically limit flexion, the model was only developed for flexion. However, the principles described here may be applied directly to develop similar models for other spinal segments or planes of motion.

## Conclusions

The parametric model permitted efficient iteration of design parameters for an implant to address flexion instability. The destabilization modeled here simulated degenerative pathology of the segment associated with DDD and DS, as well as iatrogenic destabilization associated with direct decompression. This destabilization resulted in decreased flexion stiffness and increased segmental flexion ROM and translation. The FRSS applied to destabilized segments restored ROM, stiffness and translation to intact levels, and these effects were consistent with the parametric model used to develop the FRSS. Flexion and sagittal translation have previously been shown to be coupled, and in this experiment we found that this relationship remains consistent through destabilization and re-stabilization with a flexion-restricting implant.
